# A conserved WXXE motif is an apical delivery determinant of ABC transporter C subfamily isoforms

**DOI:** 10.1247/csf.22049

**Published:** 2023-01-25

**Authors:** Md Shajedul Haque, Yoshikazu Emi, Masao Sakaguchi

**Affiliations:** 1 Graduate School of Science, University of Hyogo, Harima Science Park City, Hyogo 678-1297, Japan

**Keywords:** apical plasma membrane, sorting, ATP-binding cassette transporter, CFTR, MRP4

## Abstract

ATP-binding cassette transporter isoform C7 (ABCC7), also designated as cystic fibrosis transmembrane conductance regulator (CFTR), is exclusively targeted to the apical plasma membrane of polarized epithelial cells. Although the apical localization of ABCC7 in epithelia is crucial for the Cl^–^ excretion into lumens, the mechanism regulating its apical localization is poorly understood. In the present study, an apical localization determinant was identified in the N-terminal 80-amino acid long cytoplasmic region of ABCC7 (NT80). In HepG2 cells, overexpression of NT80 significantly disturbed the apical expression of ABCC7 in a competitive manner, suggesting the presence of a sorting determinant in this region. Deletion analysis identified a potential sorting information within a 20-amino acid long peptide (aa 41–60) of NT80. Alanine scanning mutagenesis of this region in full-length ABCC7 further narrowed down the apical localization determinant to four amino acids, W^57^DRE^60^. This WDRE sequence was conserved among vertebrate ABCC7 orthologs. Site-directed mutagenesis showed that W^57^ and E^60^ were critical for the apical expression of ABCC7, confirming a novel apical sorting determinant of ABCC7. Furthermore, a WXXE motif (tryptophan and glutamic acid residues with two-amino acid spacing) was found to be conserved among the N-terminal regions of apically localized ABCC members with 12-TM configuration. The significance of the WXXE motif was demonstrated for proper trafficking of ABCC4 to the apical plasma membrane.

## Introduction

Epithelial cells form a boundary between different extracellular environments and eventually exhibit polarity by differentiating their plasma membranes into apical and basolateral domains. The apical surface faces the lumen of tubular or sac-shaped organs such as digestive tracts and glands, and thereby forms an interface for exchange of nutrients or ions between external environment and internal tissues (Mellman and Nelson, 2008). Cystic fibrosis transmembrane conductance regulator (CFTR) was originally identified as a product of a gene mutated in patients with cystic fibrosis (CF). Initial structural analysis revealed that CFTR is a member of the ATP-binding cassette (ABC) transporter C subfamily, and also designated as ABCC7 (Riordan *et al.*, 1989). Its structure contains two membrane-spanning domains, each of which consists of six transmembrane (TM) segments, and hence classified as a 12-TM configuration transporter. Subsequent studies showed that ABCC7 is located at the apical plasma membrane of epithelial cells, where it acts as an ATP-gated and c-AMP-regulated Cl^–^ channel. Vectorial movement of Cl^–^ from epithelial cells into lumens creates an osmotic gradient, which causes passive movement of fluid from the cells to the luminal space; therefore the Cl^–^ secretion is essential for maintenance of the viscosity of the fluid covering epithelial tissues (Saint-Criq and Gray, 2017). When the Cl^–^ secretion does not work correctly, mucus in various organs becomes thick and sticky; such dysfunction of Cl^–^ secretion causes CF, which is a fatal autosomal recessive genetic disorder found frequently among Caucasian (Riordan *et al.*, 1989). ABCC7 plays a critical role in regulation of fluid and electrolyte secretion from epithelia into the respiratory tract, gastrointestinal tract, and sweat glands (Sheppard and Welsh, 1999; Aleksandrov *et al.*, 2007). Despite its physiological importance, molecular machinery responsible for the apical expression of newly synthesized ABCC7 in polarized epithelial cells is poorly understood.

Accumulating evidences emerging from recent studies have shown that the activity of ABCC7 is regulated by transcriptional, translational and post-translational mechanisms. Of these, correct localization of ABCC7 to the apical plasma membrane is of fundamental physiological importance (Amaral, 2005; Tang *et al.*, 2011). The current understanding of maintenance of intracellular trafficking of the newly synthesized ABCC7 from the endoplasmic reticulum (ER) to the plasma membrane is largely based on analyses of CF-causing mutant proteins by using cultured nonpolar cells. For example, ΔF508 is the most common mutation in CF and lacks phenylalanine at position 508. ΔF508 fails to exit the ER because of its protein folding defect leading to premature degradation by the ER quality control. However, this only accounts for how newly synthesized ABCC7 exits from the ER, while the mechanisms whereby ABCC7 that have exited from the ER moves to the plasma membrane in a polarized manner remain largely unknown.

In polarized epithelial cells, newly synthesized plasma membrane proteins are sorted into distinct carrier vesicles at the trans-Golgi network (TGN) and asymmetrically distributed into the apical or basolateral plasma membrane (Rodriguez-Boulan and Musch, 2005; Fölsch *et al.*, 2009). In addition, the plasma membrane is also involved in rapid endocytosis and recycling (Matter and Mellman, 1994). To accomplish these complicated process properly, a complex array of protein-protein interactions governs various regulatory aspects related to the polarized trafficking of proteins in epithelial cells, and thus, they contain sorting information that specifies their localization (Mellman and Nelson, 2008; Gonzalez and Rodriguez-Boulan, 2009; Weisz and Rodriguez-Boulan, 2009). Generally, strategies to identify the localization determinants of many polytopic membrane proteins have applied sequential truncation, substitution of specific amino acid residues, and construction of hybrid proteins. However, unfolded or incompletely assembled recombinant membrane proteins are often trapped in the ER, and subsequently destroyed by the ER-associated quality control mechanisms. Actually, the CF-causing ΔF508 mutation produces misfolded ABCC7 proteins (Bertrand and Frizzell, 2003). Thus, deletion or amino acid substitution introduced into the primary sequence of ABCC7 could potentially become a compromising determinant for the precise intracellular trafficking of the modified protein because of its unexpected retention in the ER.

We began this study by postulating that ABCC7 proteins, which have entered the ER and are destined for the Golgi apparatus and eventually the plasma membrane, might display localization determinants on their cytosolic surfaces, and that the cellular machinery would recognize such determinants to control delivery of ABCC7 to the apical plasma membrane. To avoid the unfavorable effects that could compromise the trafficking of genetically modified ABCC7, we set up a nondestructive assay in which the apical localization of full-length ABCC7 was disturbed under competitive conditions by overproducing a certain domain of ABCC7. Practically, such nondestructive competition assays enabled us to identify an apical targeting signal (ATS) that determined the localization of ABCC2 (Emi *et al.*, 2012). In the present study, we used this competition assay and identified an apical localization determinant within the N-terminal part (NT) of ABCC7. We further analyzed this region by alanine scanning mutagenesis and found that four amino acids, W^57^DRE^60^, comprise an important apical localization determinant for ABCC7 in polarized HepG2 cells and MDCK cells. We also found a WXXE motif to be conserved in the NTs of particular ABCC subfamily members, with a 12-transmembrane helix configuration, including ABCC4, ABCC5, ABCC11, ABCC12, and ABCC13. The significance of the WXXE motif was then demonstrated for proper trafficking of ABCC4 to the apical plasma membrane.

## Materials and Methods

### Construction of plasmids

cDNA species were amplified by reverse transcription (RT)-PCR. RT was carried out using a ReverTra Ace-α^®^ kit (TOYOBO, Osaka, Japan), and PCR was carried out using KOD-plus DNA polymerase (TOYOBO) according to the manufacturer’s instructions. ABCC7 cDNA was isolated from human testis total RNA (Takara Bio USA, Mountain View, CA, USA). The primers used for amplification are as follows: C7-F, 5'-CGCGTCGACCATGCAGAGGTCGCCTCTG-3' (the recognition site of SalI is underlined); C7-R, 5'-GGGGTACCGCGGCCGCTAAAGCCTTGTATCTTGCACC-3' (the NotI site is underlined). The amplified DNA fragment containing the entire coding sequence was treated with NotI plus SalI and ligated into p3xFLAG-CMV-10 (Sigma, St. Louis, MO, USA). ABCC4 cDNA was isolated from human liver total RNA (Takara Bio USA). The primer sequences are as follows: C4-F, 5'-CCGAAGCTTATGCTGCCCGTGTACCAGGAG-3' (the HindIII site is underlined); C4-R, 5'-GCCGAATTCTCACAGTGCTGTCTCGAAAATAG-3' (the EcoRI site is underlined). The amplified DNA fragment was treated with HindIII plus EcoRI and ligated into p3xFLAG-CMV-10.

Site-directed *in vitro* mutagenesis of ABCC7 and ABCC4 was carried out using the QuickChange kit (Agilent, Santa Clara, CA, USA). The primers used for creating the site-directed mutants are listed in supplementary Table SI. A coding sequence encompassing the NT80 region of ABCC7 was amplified by PCR using the C7-F and the following primer: C7-NTR, 5'-CGGGGTACCTATCTCCAGAAAAAACATCGCCGAAGG-3' (KpnI restriction site is underlined).

The amplified DNA fragment was digested with SalI plus KpnI and the digested fragment was then cloned into the pCMV-Myc (Clontech, Palo Alto, CA, USA) to generate a Myc-NT80 fusion protein. Stepwise deletion of the Myc-NT80 coding DNA fragment was performed using an inverse PCR-based site-specific mutagenesis method (Wang and Wilkinson, 2001). The primers used for creating these deletions are listed in supplementary Table SI. The nucleotide sequences of the mutants were confirmed by dideoxy sequencing of the respective DNA fragments.

### Expression of recombinant proteins in HepG2 cells

HepG2 cells and MDCK cells were maintained in Dulbecco’s modified Eagle’s medium supplemented with 10% fetal calf serum under an atmosphere of 5% CO_2_ at 37°C. Cells were transiently transfected using PEI-MAX reagent (Polysciences, Warrington, PA, USA) as described (Boussif *et al.*, 1995; Longo *et al.*, 2013). Briefly, 2.0 × 10^5^ cells were seeded into a 3.5-cm dish and cultured for 24 h. We use a 5:1 ratio of PEI to DNA (w/w) for preparation of a DNA-PEI complex. Cells were incubated with the DNA-PEI complex for 24 h, washed once with prewarmed culture medium, and further incubated in fresh medium. Transfected cells were harvested, disrupted in SDS-PAGE sample buffer by sonication, and centrifuged at 15,000 rpm for 5 min to remove cell debris. The clarified supernatant was subjected to immunoblot analysis using either anti-Myc monoclonal antibody (Covance, Princeton, NJ, USA) or anti-FLAG monoclonal antibody (Sigma-Aldrich, St. Louis, MO, USA). Immunoreactive protein bands were probed with peroxidase-conjugated goat anti-mouse IgG (Covance) and visualized using ECL reagent (Amersham Biosciences, Piscateway, NJ, USA) and an LAS1000 image analyzer (Fuji, Tokyo, Japan).

### Analysis of subcellular localization of FLAG-CFTR and FLAG-ABCC4

Immunofluorescence studies were carried out essentially as described previously (Tsuchida *et al.*, 2008; Emi *et al.*, 2011). LAMP1-RFP (Addgene plasmid #1817) was a gift from Walther Mothes (Sherer *et al.*, 2003). mCherry-Sec61β (Addgene plasmid #121160) was a gift from Christine Mayr (Ma and Mayr, 2018). DsRed-Rab5 WT (Addgene plasmid #13050), DsRed-rab7 WT (Addgene plasmid #12661), and DsRed-rab11 WT (Addgene plasmid #12679) were supplied from Richard Pagano (Choudhury *et al.*, 2002; Sharma *et al.*, 2003). To detect the epitope-tagged proteins by immunostaining, transfected cells were incubated sequentially with anti-FLAG monoclonal antibody (1:5,000 dilution) for 1 h and then with Alexa Fluor 488-conjugated anti-mouse IgG (1:5,000 dilution; Thermo Fisher Scientific, Waltham, MA, USA) for 1 h. Immunofluorescence was observed using a confocal laser scanning microscope (LSM5-PASCAL; Carl Zeiss, Jena, Germany) equipped with a multitrack configuration specified in the Zeiss Aim software. Fluorescent images were observed through an LP 650 filter and a BP 505–530 filter by sequential excitation with 633 nm and 488 nm laser beams, respectively.

**Fig. 1:**
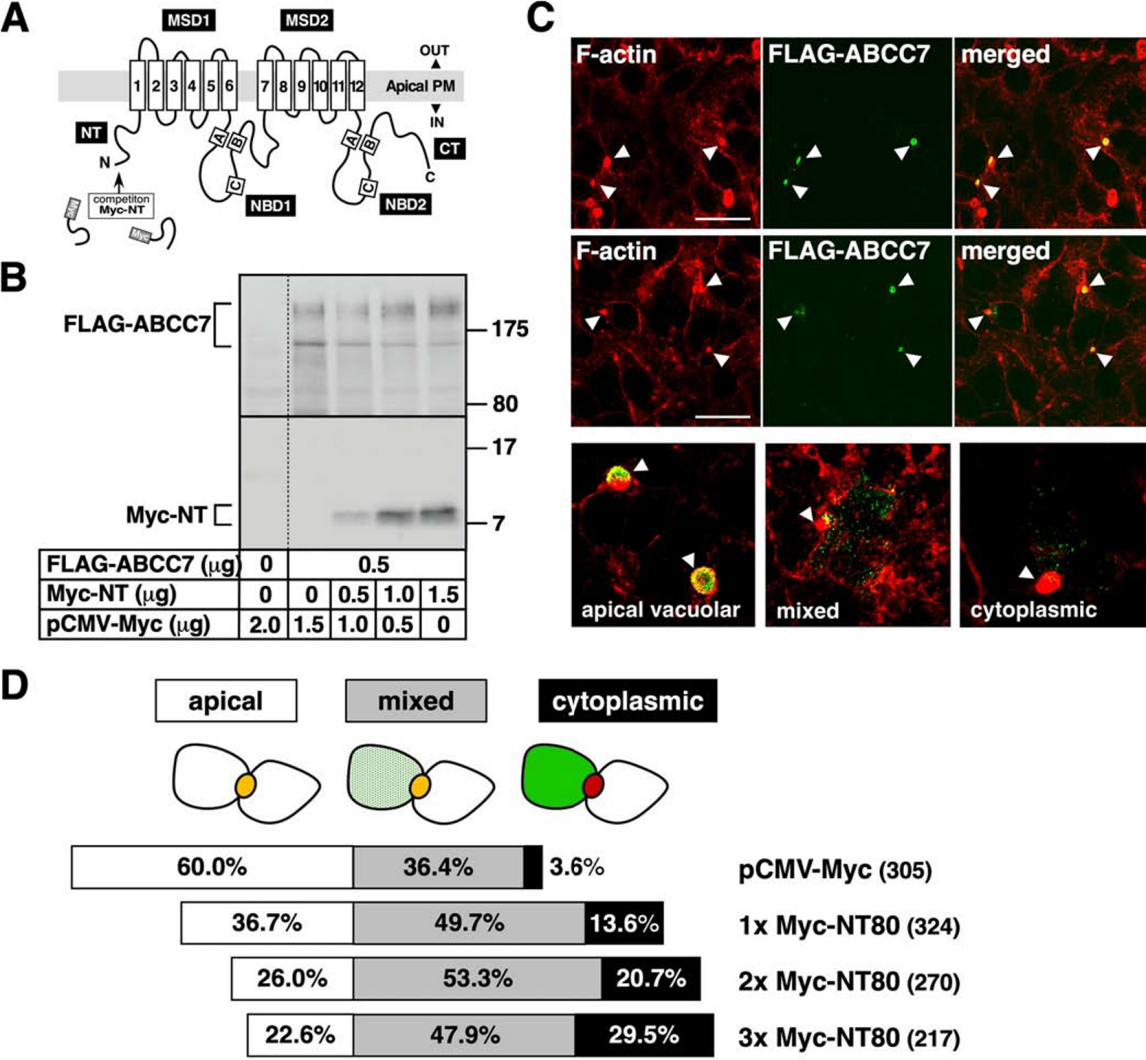
Disruption of the apical localization of ABCC7 by overexpression of NT80 from ABCC7 (A) Predicted membrane topology of the human ABCC7 and a scheme depicting the possible competitive effect of overproduced NT80. This membrane topology prediction was carried out using the TMpred program (http://www.ch.embnet.org/software/TMPRED_form.html). Walker A and B motifs and the family signature C are also indicated (white letters). (B) Expression of FLAG-ABCC7 and Myc-NT80 in HepG2 cells. The indicated combinations of FLAG-ABCC7 and Myc-NT80 were used to transiently transfect HepG2 cells. The total amount of plasmid DNA used for each transfection was kept the same (2 μg) by including the empty vector pCMV-Myc whenever needed. The blot shown is representative of three independent experiments. The positions of the protein molecular weight markers are indicated on the right hand side of the blot. (C) Effect of overexpression of NT80 on the localization of FLAG-ABCC7 in HepG2 cells. Cells were transiently co-transfected with FLAG-ABCC7 (0.5 μg) and Myc-NT80 (1.0 μg) expression plasmids. Canalicular vacuoles and cell perimeters were visualized with rhodamine-conjugated phalloidin (F-actin). Shown are the representative immunofluorescence images. Three typical localization are shown in the bottom panels. Scale bars: 20 μm. (D) Quantification of the disruption of apical localization of ABCC7 by overexpression of NT80. HepG2 cells were cotransfected with the FLAG-ABCC7 plasmid and the indicated competitor (empty or Myc-NT80) plasmid and localization of ABCC7 was determined in these cells as described in (C). For each cell, the degree of colocalization of FLAG-ABCC7 integrated into the apical vacuole was categorized into three groups as illustrated. The yellow area represents co-localization that is most similar to the normal condition. The percentage of cells displaying each localization pattern was plotted (horizontal bar plot); Open bar, apical; light gray-shaded bar, intermediate; and dark gray-shaded bar, cytoplasmic. Polarized cells were counted according to a criteria based on the formation of the apical vacuole, and the number of cells is shown in parentheses.

To assess polarity, transfected cells were stained with rhodamine-conjugated phalloidin (Thermo Fisher Scientific). Apical vacuoles are formed between adjacent polarized HepG2 cells and are visualized by using rhodamine-conjugated phalloidin. Polarized cells were counted according to a criteria that relied on the formation of sealed apical vacuoles between the neighboring cells. To circumvent the unfavorable effects that could arise from the overexpression of proteins, reduced amounts of expression plasmids (typically 0.5 μg) were used for transient transfection, and polarized cells exhibiting dense staining of FLAG-CFTR throughout their cytoplasm were excluded from the localization analysis. For each transfection, polarized cells (as observed by rhodamine-decorated punctate fluorescence) expressing FLAG-CFTR (as observed by Alexa Fluor 488 fluorescence) were examined. Three independent transfections were performed and cells were analyzed 48 h after transfection. The degree of co-localization of FLAG-CFTR with the apical vacuoles was categorized into one of the following three groups as described previously (Emi *et al.*, 2011, 2012). Briefly, “apical vacuolar” localization of FLAG-CFTR was defined as the fluorescent signal from FLAG-CFTR forming a single large punctuated staining that completely merged with that from the apical vacuoles (*see* Fig. 1). When FLAG-CFTR was virtually absent from the apical vacuoles in polarized cells, the distribution was defined as “cytoplasmic”. “Intermediate” localization refers to partial co-localization, where the FLAG-CFTR was distributed to the apical membrane as well as to other intracellular locations.

## Results

### Confirmation of apical expression of FLAG-ABCC7 in HepG2 cells

HepG2 cells are structurally and functionally polarized. In monolayer culture, a sealed vacuole is formed between the plasma membranes of two adjacent polarized cells. The plasma membranes of the vacuoles of HepG2 cells in monolayer culture (Sormunen *et al.*, 1993) and the bile canaliculi of hepatocytes in liver tissues (Arias *et al.*, 2020) represent the apical domain. The apical vacuoles and cell perimeters are readily visualized in polarized HepG2 cells under a microscope as fluorophore-conjugated phalloidin-derived signals (van der Wouden *et al.*, 2002); thus, such vacuoles and cell perimeters seen in HepG2 cells are convenient indicators of apical and basolateral domains, respectively. Actually, we previously showed that a monolayer culture of HepG2 cells can serve as a useful model system to study the polarized localization of ABCC1 and ABCC2 (Emi *et al.*, 2012, 2013). ABCC7 is sorted to the apical plasma membrane of polarized epithelial cells. Like FLAG-ABCC2, immunostaining of FLAG-ABCC7 produced a single punctuated signal between the neighboring HepG2 cells, which merged with the apical vacuoles (supplementary Fig. S1B). This result suggested that fusion of the FLAG-tag to ABCC7 did not affect its localization, as this fusion protein was targeted in a manner identical to that of its endogenous counterpart. This result also suggested that a monolayer culture of HepG2 cells can be used to study the polarized distribution of ABCC7.

### Apical localization information resides in the N-terminal 80-amino acid long cytoplasmic region of ABCC7

ABCC subfamily members typically comprise two tandemly-arranged polytopic membrane spanning domains of six transmembrane (TM) helices (MSD1 and MSD2) and two cytoplasmic nucleotide binding domains (NBD1 and NBD2) (Dean *et al.*, 2001; Deeley *et al.*, 2006). ABCC7 and ABCC2 are related members of the ABCC subfamily that display a comparable membrane topology (supplementary Fig. S1A). Based on the numbers and the topology of transmembrane regions, the ABCC subfamily members fall into two groups. ABCC7 is one of the 12-TM configuration member harboring two MSDs. Notably, the N-termini of the 12-TM configuration species are located in the cytosol. While 17-TM configuration members, such as ABCC2, have an extracellularly located N-terminal and an additional MSD0 region which contains five TM helices. Moreover, MSD0 exists at the N-terminus of ABCC2 and the first cytoplasmic loop domain (CLD1) connects MSD0 to MSD1. We previously revealed that an apical targeting sequence (ATS) within the CLD1 is required for proper apical targeting of ABCC2 in polarized cells (Emi *et al.*, 2012). Like CLD1 of ABCC2, an N-terminal 80-amino acid long cytoplasmic region of ABCC7 (NT80) is followed by MSD1. However, physiological significance of NT80 still remains elusive; we thus postulated that the NT80 may be involved in the apical expression of ABCC7.

Many proteins that are trafficked from the ER to the plasma membrane might display sorting determinants on their cytosolic surface, and the cellular trafficking machinery would recognize such determinants to navigate them (Altschuler *et al.*, 2003; Gonzalez and Rodriguez-Boulan, 2009; Weisz and Rodriguez-Boulan, 2009). Formerly, we set up a nondestructive assay in which the apical localization of full-length ABCC2 was disturbed under competitive conditions by overproducing CLD1. Initially, we postulated that overexpression of NT80 would compete the apical localization of ABCC7 (Fig. 1A). To explore this possibility, we constructed an expression plasmid in which a Myc-tag was fused to the N-terminus of NT80 (Myc-NT80); then increasing amounts of Myc-NT80 plasmid were mixed with a constant amount of FLAG-ABCC7 plasmid and the mixtures were used separately in a competition assay by transient transfection of HepG2 cells. Immunoblot analysis revealed a single band of Myc-NT80 with an apparent molecular weight of 8,000 and the intensity of this protein band increased with increasing amounts of the Myc-NT80 plasmid, while co-expressed FLAG-ABCC7 remained roughly the same in all cases (Fig. 1B).

For initial competition study, we chose to use 1.0 μg of Myc-NT80 plasmid and 0.5 μg of FLAG-ABCC7 plasmid to transfect HepG2 cells. As shown in Fig. 1C and supplementary Fig. S1C, in addition to their normal “apical vacuolar” localization, the expressed FLAG-ABCC7 showed a “cytoplasmic” distribution and “mixed” distribution with reduced apical localization. In each FLAG-ABCC7-expressing polarized HepG2 cell, the intracellular distribution of FLAG-ABCC7 seemed to varied roughly depending on levels of Myc-NT80 expression seen by immunofluorescent staining (supplementary Fig. S1D). Normal apical localization of FLAG-ABCC7 was predominantly observed in the cells which express no or the lesser Myc-NT80 expression. By contrast, accumulation of cytoplasmic Myc-NT80 reduced the apical vacuolar localization of FLAG-ABCC7 with reciprocal increase in the intracellular accumulation of mislocalized FLAG-ABCC7 accompanied by appearance in the dot-like structures. The observed dot-like signals partially overlapped with Rab5-positive early endosome as well as Rab11-positive recycling endosome, but did not with Sec61β-positive ER, LAMP1-positive lysosome, and Rab7-positive late endosome (supplementary Fig. S2B).

For quantification, the degree of co-localization of FLAG-ABCC7 integrated into the apical vacuoles was categorized into one of three groups (Fig. 1D). Consistently, co-expression of increasing amounts of Myc-NT80 over that of FLAG-ABCC7 significantly reduced the “apical vacuolar” localization of ABCC7, with a reciprocal increase in “mixed” and “cytoplasmic” distribution of mislocalized ABCC7, leading to a significant number of cells exhibiting “cytoplasmic” distribution. The “mixed” distribution of ABCC7 could have resulted from the defective integration of these proteins into the apical vacuoles, as it appeared in both the apical vacuoles and dot-like appearances. In contrast, co-transfection with excess empty pCMV-Myc did not have any effect on the apical localization of FLAG-ABCC7. These observations suggested that overproduction of Myc-NT80 disturbed the polarized distribution of full-length ABCC7 in a competitive manner, thereby indicating that apical localization information might be present in the NT80 of ABCC7. In contrast, co-expression of the CLD1 polypeptides, which derived from ABCC1 (C1-CLD1) or ABCC2 (C2-CLD1), failed to interfere the apical expression of FLAG-ABCC7, which was similar to that observed in the control cells (supplementary Fig. S3). These findings also suggested that a saturable sorting machinery for ABCC7 could present within polarized HepG2 cells.

### Identification of an apical localization determinant for ABCC7

**Fig. 2:**
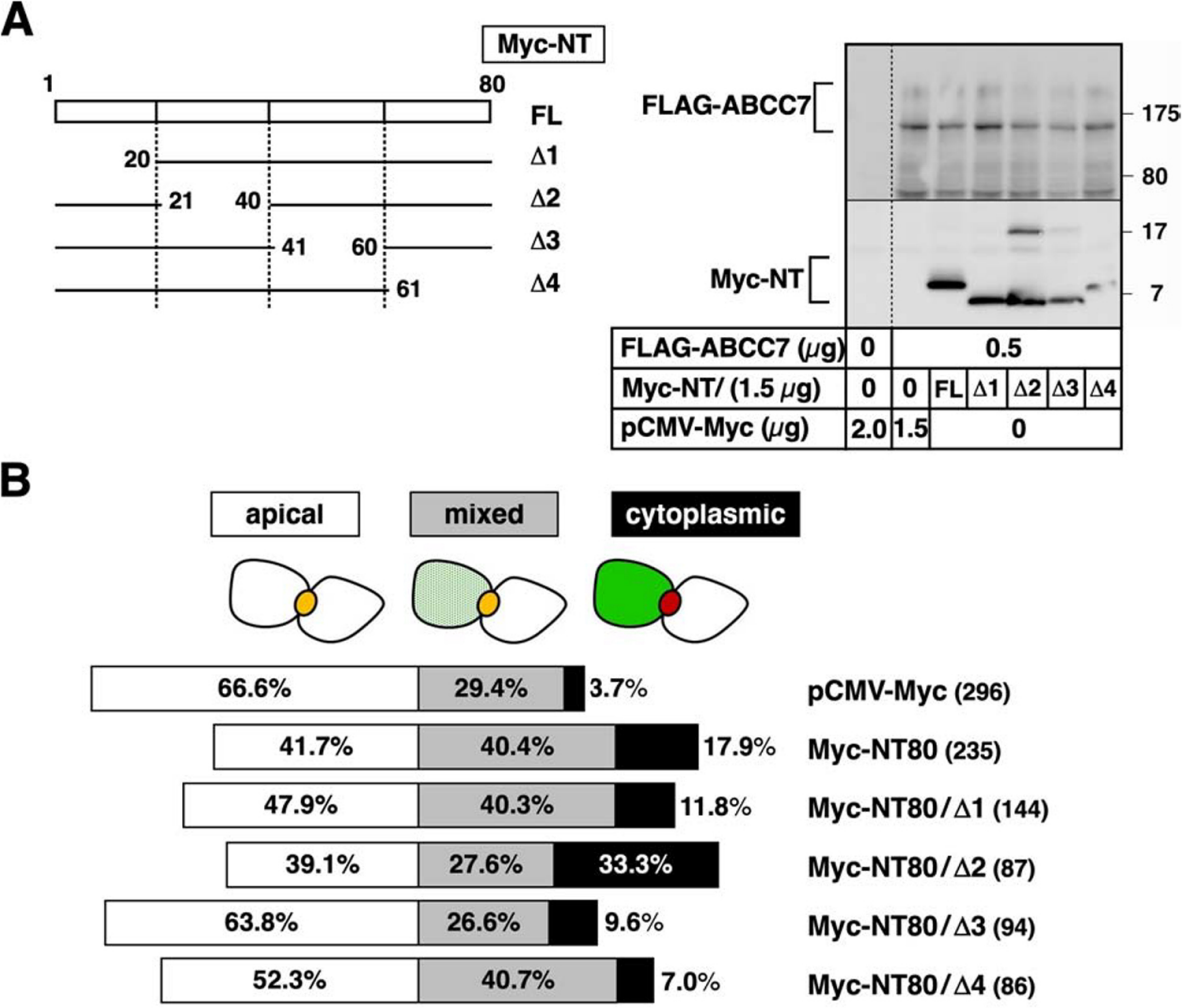
Assignment of a functional element in NT80 responsible for the apical expression of ABCC7 (A) Confirmation of transient expression of ABCC7 and successively deleted NT80s in HepG2 cells. Illustration (on the left) showing the Myc-NT80 construct (FL) and its deletion derivatives (Δ1–Δ4) created in this study. Numbers shown beside each construct indicate the deletion points from the translation start site of ABCC7. HepG2 cells were transiently transfected with 1.5 μg of the indicated CLD1 deletion plasmid and 0.5 μg of FLAG-ABCC7 plasmid. The blot shown here (right panel) is representative of three independent experiments. The positions of protein molecular weight markers are indicated on the left. (B) Disruption of apical localization of ABCC7 by overexpression of a series of deleted NT80. HepG2 cells were cotransfected with FLAG-ABCC7 and the indicated competitor (FL or NT80 deletion) plasmid and localization of FLAG-ABCC7 was determined as described previously. The degree of colocalization of FLAG-ABCC7 integrated into the apical vacuole of each cell was categorized as in Fig. 1. All assays were performed in triplicate and the percentage of cells displaying each type of localization was plotted (horizontal bar plot). Polarized cells were counted according to a criteria based on the formation of the apical vacuole, and number of cells is shown in parentheses.

To identify the apical localization determinant(s) of ABCC7, we created a series of Myc-NT80 truncations, each carrying a deletion of approximately 20 residues (Fig. 2A). We then analyzed the effects of overexpression of these NT80 deletion mutants on the apical localization of FLAG-ABCC7. As shown in Fig. 2B, co-expression of the Δ3 deletion mutant of NT80, which lacked amino acid residues 41–60 of ABCC7, failed to perturb the apical localization of FLAG-ABCC7. In contrast, co-expression of the other three deletion mutants (Δ1, Δ2, and Δ4) disturbed the polarized distribution of FLAG-ABCC7 in HepG2 cells roughly to the same extent. These results suggested that the peptide region, consisting of amino acid residues 41–60 of ABCC7, harbored a determinant for apical localization of CFTR.

**Fig. 3:**
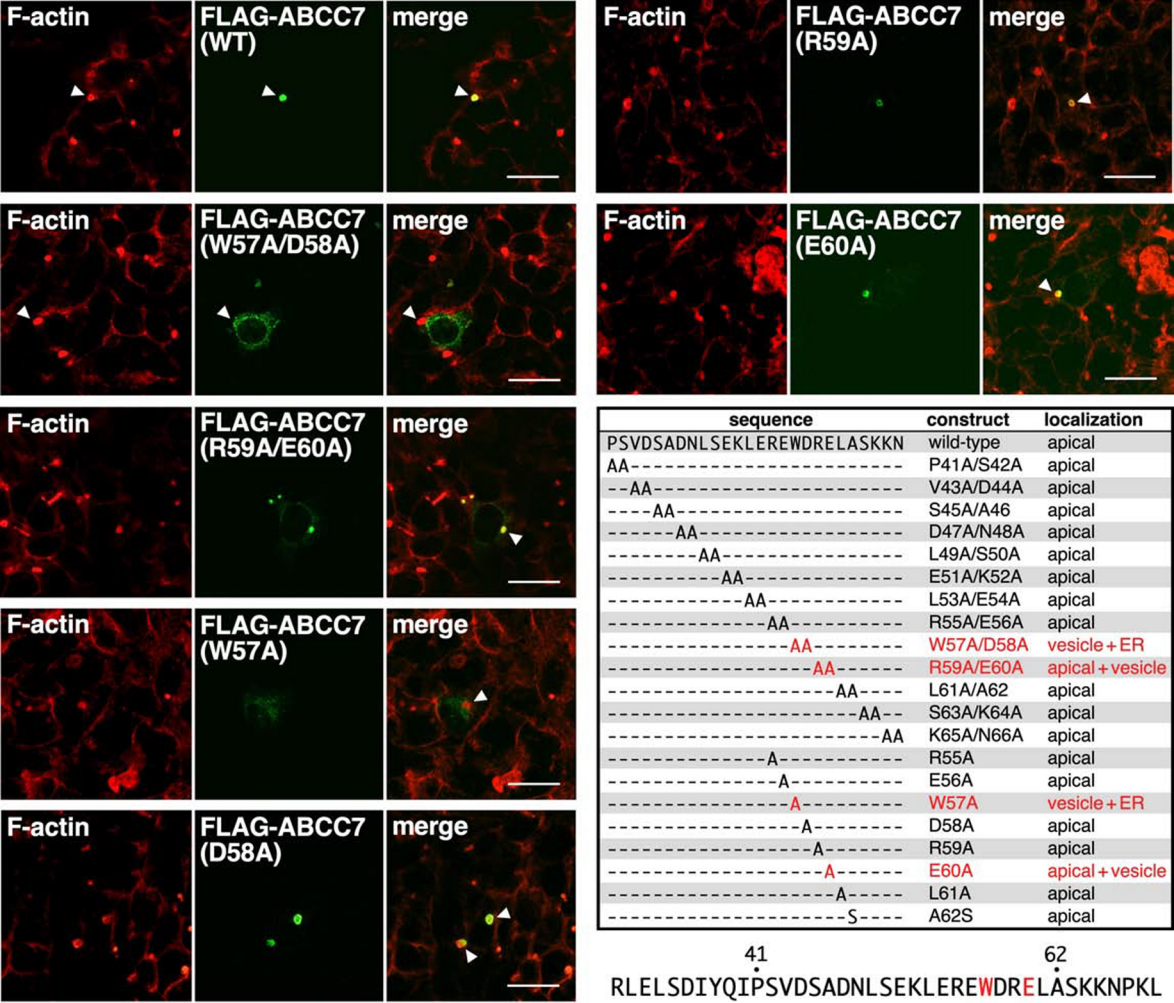
Identification of an apical expression determinant in a four-amino acid motif of ABCC7 Residues 41–66 in the NT80 region of ABCC7 were subjected to alanine scanning mutagenesis as indicated. Localization of each mutant in polarized HepG2 cells was carried out as described before and the results summarized. HepG2 cells were transiently cotransfected with 0.5 μg of the indicated FLAG-ABCC7 expression plasmids along with 0.5 μg of empty vector pCMV-HA. Forty hours after transfection, cells were fixed and processed for indirect immunofluorescence analysis. Shown are the representative immunofluorescence images of wild-type FLAG-ABCC7, two di-alanine substitution mutants of ABCC7 (W57A/D58A and R59A/E60A), and four single-alanine substitution mutants of ABCC7 (W57A, D58A, R59A, and E60A). Apical vacuoles formed between two juxtaposed cells (indicated by white arrowheads) were visualized with rhodamine-conjugated phalloidin (F-actin). Scale bars: 20 μm. Results of intracellular localization of FLAG-tagged alanine-scanning mutants in polarized HepG2 cells are summarized in supplementary Fig. S3.

We next focused on this stretch of the 20-amino acids and its neighboring residues of NT80. Accordingly, we generated 13 substitution mutants of FLAG-ABCC7 by successive substitution of every two amino acid residues within this region of FLAG-ABCC7 with di-alanine as indicated in Fig. 3. The intracellular distribution of the di-alanine scanning mutants of FLAG-ABCC7 were then examined by transiently expressing them in HepG2 cells (supplementary Fig. S4). Remarkably, two of the di-alanine scanning mutants, W57A/D58A and R59A/E60A, showed perturbed localization patterns accompanied with reticular and dot-like signals. Especially, the R59A/E60A mutant showed mixed staining patterns (staining of apical vacuoles as well as the dot-like structures). On the other hand, transient expression of the other 11 mutants of flanking regions resulted in normal apical localization, similar to that of the wild-type FLAG-ABCC7. These results lowered the possibility that perturbed localization of the W57A/D58A and R59A/E60A mutants was caused by gross protein misfolding, which could inhibit trafficking of ABCC7 to the apical membrane. Taken together, our results suggested that the four-amino acid stretch, W^57^DRE^60^, harbors important information for the apical localization of human ABCC7 in polarized HepG2 cells.

### W^57^ and E^60^ are critical residues for the apical localization of ABCC7

Next, we determined the relative significance of individual residues within this W^57^DRE^60^ stretch and flanking residues on either side in terms of the apical localization by analyzing the distribution of the eight FLAG-ABCC7 mutants harboring alanine substitutions at each position (Fig. 3 and supplementary Fig. S5). In particular, two point mutants, W57A and E60A, exhibited impaired localization patterns accompanied with reticular and dot-like appearance. These signals overlapped partially with mCherry-Sec61β and DsRed-Rab5 (supplementary Fig. S5A). The other point mutants including D58A and R59A, exhibited only wild-type like apical localization. These results repeatedly exclude the possibility that impaired distribution of W57A and E60A mutants was caused by gross protein misfolding. We next generated additional point mutations at either W^57^ or E^60^ in FLAG-ABCC7 to establish the contribution of each to the apical localization of ABCC7. Notably, W57G and E60K are CF-causing mutations, each of which causes CF when combined with a different CF-causing mutation (CFTR2; https://cftr2.org/). Ectopically expressed W57G and E60K mutants were found to be essentially associated with ER marker proteins (supplementary Fig. S5B). By contrast, several mutants, such as W57F, W57R, E60Q, and E60V, exhibited less severely impaired delivery to the apical vacuoles (supplementary Fig. S5C).

**Fig. 4:**
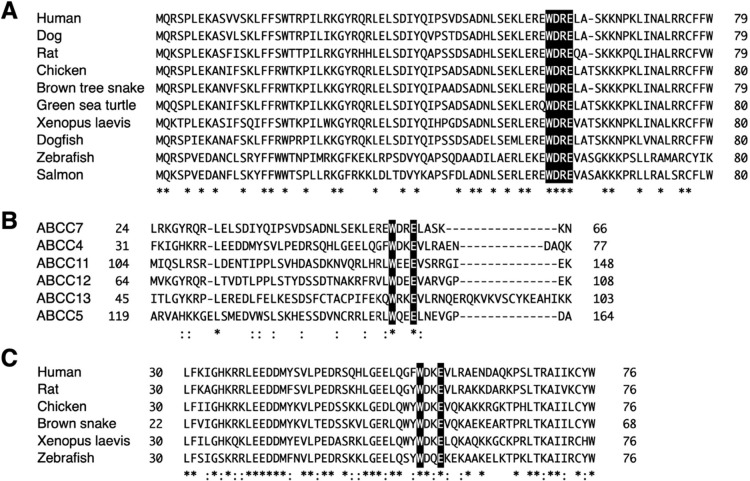
Identification of a WXXE motif as an apical expression determinant among ABCC subfamily members with the 12-TM configuration (A) Comparison of NT regions from various species. The 79 amino acid NT region of human ABCC7 (M28668) was compared with the identical regions within ABCC7 homologs from dog (AY780429), rat (M89906), chicken (XP_015131458), snake (JU174160), turtle (KB478633), clawed frog (X65256), dogfish (M83785), zebrafish (BC171654), and salmon (NP_001117006). Residues identical to those found in the human ABCC7 sequence are highlighted by white letters. Asterisks indicate identical residues in all species. (B) Amino acid sequence alignment of NT regions from ABCC isoforms with the 12-TM configuration. The 43-amino acid long NT region of human ABCC7 was compared with the identical regions from ABCC4 (AF071202), ABCC11 (AF367202), ABCC12 (AF395908), ABCC13 (AY340665), and ABCC5 (AF104942). Conserved tryptophan and glutamic acid residues are highlighted in white letters. Asterisk and colon indicate identical residues and conserved residues, respectively. (C) Comparison of the WXXE motif of ABCC4 from various species. The 47 amino acid NT region of human ABCC4 was compared with the identical regions within ABCC4 homologs from rat (AY533524), chicken (KU678216), snake (XP_026524765), clawed frog(XP_018102737), and zebrafish (NP_001007039).

**Fig. 5:**
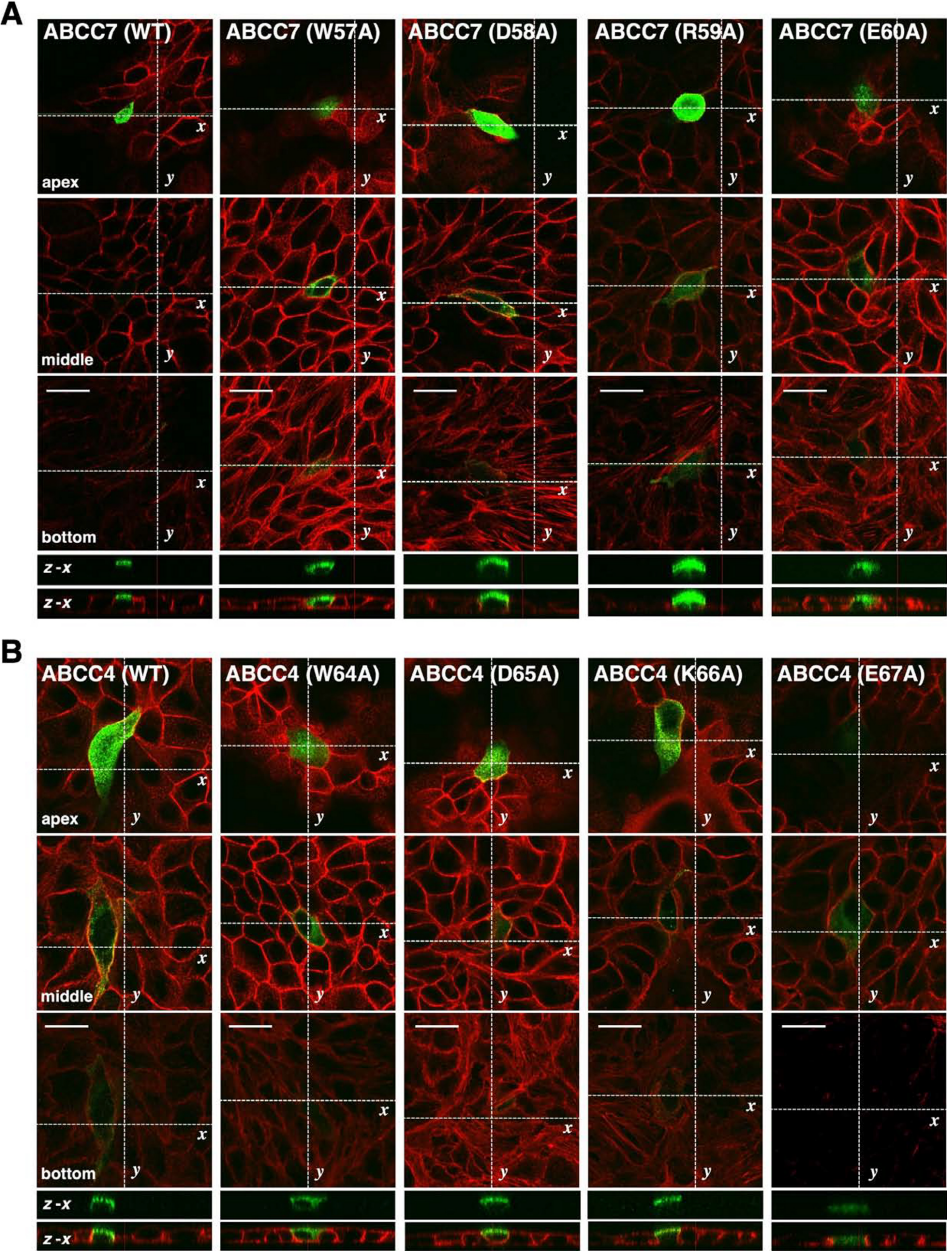
Subcellular distribution of FLAG-ABCC7 and FLAG-ABCC4 in MDCK cells (A) MDCK cells expressing wild-type ABCC7 or the indicated ABCC7 mutants were immunostained with anti-FLAG mAb (green). Cell perimeter was stained with Rhodamine-phalloidin (red) and representative merged images are shown. Single merged panels of representative apical (apex), lateral (middle), and basal (bottom) sections are shown. The W57A and E60A mutants displayed a mislocalization to the intracellular localization (*see* middle and z–x sections). (B) MDCK cells expressing wild-type ABCC4 or the indicated ABCC4 mutants were immunostained. The W64A and E67A mutants displayed a mislocalization to the intracellular localization (*see* middle and z–x sections). Scale bar, 20 μm.

As shown in Fig. 4A, the four-amino acid long WDRE sequence is completely conserved among the listed 11 vertebrate species. This high sequence conservation also suggests the involvement of tryptophan and glutamic acid residues in the apical localization of vertebrate ABCC7. To determine whether similar trafficking is observed in MDCK cells, we generated transient transfectants using the selected set of mutants (W57A, D58A, R59A, and E60A). MDCK cells are columnar epithelial cells and form the apical domain at the cell apex. As shown in Fig. 5A, FLAG-ABCC7 was seen exclusively at the apical side of MDCK cells, and the D58A and R59A mutants also displayed normal apical accumulation. In contrast, the W57A and E60A mutants lost apical-specific steering activity and gave the mixed staining of the cytosolic region in addition to the apical side. In other words, the latter two mutants showed reduced apical distribution with reciprocal increase in the cytoplasmic accumulation of mislocalized FLAG-ABCC7. Consequently, these forms displayed the same trafficking profiles observed in HepG2 cells. Taken together, we concluded that W^57^DRE^60^ is a core motif of apical localization determinant of human ABCC7, in which the residues W^57^ and E^60^ are essential for correct localization.

### Conserved WXXE motif is critical for the apical localization of ABCC subfamily members with the 12-TM configuration

In addition to ABCC7, apically localized ABCC subfamily members of 12-TM configuration are ABCC4, ABCC5, ABCC11, ABCC12, and ABCC13. Comparison of their N-terminal amino acid sequences by using Crustal revealed a conserved motif, where tryptophan and glutamic acid residues are separated by two amino acids (Fig. 4B). Hereafter we referred this conserved element as a WXXE motif. This finding raised the possibility that the WXXE motif is an essential apical localization determinant of the 12-TM configuration species. To address this possibility, we next focused on the WXXE motif of human ABCC4, W^64^DKE^67^. As shown in Fig. 4C, this WDKE sequence is conserved among vertebrate ABCC4 species (Fig. 4C). ABCC4 acts as a high affinity efflux exporter for cAMP, and spatiotemporal coupling of ABCC4 with ABCC7 modulates the Cl^–^ channel activity of ABCC7 in the apical plasma membrane of the gut epithelia (Li *et al.*, 2007). Unexpectedly, immunostaining of FLAG-ABCC4 showed cytoplasmic distribution in HepG2 cells; we thus examined its localization by using MDCK cells. As shown in Fig. 5B, FLAG-ABCC4 was readily seen at the apical side of MDCK cells as expected. This different sorting behavior of these two epithelial cell lines may be attributed to their differential interpretation of the WXXE motif and other sorting informations of ABCC4, but the underlying reason remains to be clarified. The W64A mutant lost its apical-specific trafficking activity and gave the mixed staining of the cytosolic side in addition to the apical side. Localization of E67A mutant looked mostly intracellular in MDCK cells. By contrast, the D65A and K66A mutants displayed normal apical localization. Accordingly, the WDKE sequence of ABCC4 is capable of exerting its apical steering activity. Taken together, these results indicates that the conserved WXXE motif acts as an apical localization determinant of the ABCC subfamily members of the 12-TM configuration.

## Discussion

The most important finding of this report is the identification of a four-amino acid residue long peptide sequence, WXXE motif, that is required for the apical expression of the 12-TM configuration ABCC subfamily members in polarized epithelial cells. Our findings can be summarized as follows. First, overproduction of NT80 of ABCC7 significantly disturbed the apical distribution of full-length ABCC7 in a competitive fashion. This finding encouraged us to set up a competition assay as described in a previous report, in which we identified a regulatory motif that determined the apical targeting of ABCC2 (Emi *et al.*, 2012). Generally, analysis of truncation mutants and hybrids facilitates the initial mapping of such regulatory motifs. Our nondestructive approach has a potential advantage in that it does not require the construction of genetically modified ABCC7 and ABCC4 for the initial mapping of localization determinants and, thereby, avoids the complications that are generally associated with the failure of protein folding and tertiary structure formation. Second, alanine scanning mutagenesis revealed that a regulatory core motif (W^57^DRE^60^) are critical for apical expression of ABCC7. In particular, amino acids W^57^ and E^60^ were functionally important residues. Third, two CF-causing mutations, W57G and E60K, were assigned to this regulatory motif, thereby implying its physiological importance. Fourth, a conserved WXXE motif was found in ABCC subfamily members with the 12-TM configuration that are localized to the apical plasma membrane like ABCC7. This motif was capable of steering the apical distribution of ABCC4.

To determine the relative importance of individual residues W^57^ and E^60^, we generated Myc-NT80 mutants harboring either W57A or E60A. We then analyzed the effects of overexpression of these mutant competitors on the apical localization of FLAG-ABCC7. However, we could not satisfactorily expressed these mutant proteins in HepG2 cells (data not shown). Based on the cryo-EM structure of zebrafish ABCC7 (Zhang and Chen, 2016), the WDRE core motif is situated in the lasso motif. Recently published cryo-EM–derived structures of ABCC subfamily proteins revealed the presence of the similar lasso-like structures among them (Lee and Rosenbaum, 2017). We next asked whether the WDRE motif of ABCC7 could exert its apical steering activity on basolaterally distributing ABCC2/S283A mutant (Emi *et al.*, 2012). For this purpose, the M^246^KRE^249^ peptide sequence of the ABCC2, which is located in the lasso-like domain of ABCC2, was replaced with the WDRE sequence to create ABCC2/S283A^WDRE^. Unfortunately, ABCC2/S283A^WDRE^ exhibited severely impaired delivery to the apical vacuoles, and was found to be essentially associated with ER marker proteins (data not shown); we did not find conclusive evidence to address this question.

The polarized distribution of ABCC7 is of physiological importance for maintaining the vectorial movement of Cl^–^ (Saint-Criq and Gray, 2017). The *cis*-acting localization determinants and trans-acting cellular mechanisms must interact to ensure accurate apical delivery of ABCC7, and the molecular machinery responsible for the apical localization of ABCC7 has begun to emerge. For example, several Rab GTPases have been implicated in the regulation of the intracellular trafficking to the plasma membrane. Particularly, Rab11 interacts with both ABCC7 and myosin Vb, thereby controlling vesicle transport through the actin cytoskeleton (Swiatecka-Urban *et al.*, 2007). It has been revealed that the C-terminal region of ABCC7 interacts with scaffolding adapter proteins. Of these, post-synaptic density 95/disk large/zonula occludens-1 (PDZ) family proteins, such as Na^+^/H^+^ exchanger regulating factor-1 (NHERF-1) and PDZ domain-containing kidney protein 1 (PDZK1), interact with cytoskeletal adapter proteins such as radixin and ezrin (Li *et al.*, 2010). These adapter proteins form a macromolecular complex with the F-actin cytoskeletal network underneath the subplasmalemmal domain and are considered important for the stable anchoring of the delivered ABCC7 in the apical membrane (Short *et al.*, 1998; Swiatecka-Urban *et al.*, 2002). In fact, we previously demonstrated that an interaction between the C-terminus of ABCC2 and the PDZ adapter proteins helps to stabilize the apical anchoring of ABCC2 (Emi *et al.*, 2011). Syntaxin 3 is a cell surface syntaxin family protein that is involved in apical recycling and in biosynthetic traffic from the TGN to the apical plasma membrane in epithelial cells. Recently, Syntaxin 3 was shown to physically interact with ABCC7 and play a role in the exocytic trafficking of ABCC7 (Collaco *et al.*, 2010). However, the mechanism regulating the apical expression of newly synthesized ABCC7 in polarized epithelial cells remains elusive. In this study, we present one such mechanism by demonstrating that the narrowly defined peptide sequence, W^57^DRE^60^, is important for apical localization of ABCC7.

How does the WXXE motif guide apical targeting of ABCC7 and ABCC4? Modified ABCC7 and ABCC4 constructs lacking the functional WXXE motif were not sorted properly. It is noteworthy that such mutants were observed at both the endosomal compartments along with the apical plasma membranes. This suggests that the WXXE motif is probably one of the determinants essential for directing the apical localization of ABCC7 and ABCC4, but not required for proper protein folding and assembly. However, other as-yet-unidentified regions may contain auxiliary information regarding its proper apical localization, and they appear to become functional in the native conformation of ABCC7 and ABCC4, where several motifs may combine. The newly synthesized ABCC7 and ABCC4 communicates with the apical sorting machinery. It remains to be determined how the WXXE motif is interpreted and what molecular machinery interacts with this motif. Based on our results, we cannot conclusively suggest that this motif interacts with a cellular component such as sequence-specific binding protein, however, our data suggest the existence of a saturable sorting machinery because the polarized distribution of ABCC7 was disturbed by overexpression of NT80 in a competitive manner. Defining the protein(s) that binds to ATS should provide insights into the nature of the molecular mechanism of polarized distribution of physiologically important transporters in epithelial cells. To this end, we are currently performing affinity pull-down experiments and two-hybrid screening to identify binding proteins, which might provide valuable clues to the mechanism of polarized protein delivery in epithelial cells.
