# Supplementary material for: A conserved WXXE motif is an apical delivery determinant of ABC transporter C subfamily isoforms
Source: Cell Struct Funct. 2023 Jan 25;48(1):71–82. doi: 10.1247/csf.22049 (PMC10721954; doi:10.1247/csf.22049)
Supplement: Supplementary file 1 — Supplementary Table [file csf_48_22049_1.pdf]

Supplementary material

Table SI. Sequences of oligonucleotide primers used for *in vitro* site-directed mutagenesis.

construction name (primer sets)	sequence (5' to 3')	
	forward primer	reverse primer
**1. Alanine scanning mutagenesis (QuickChange): ABCC7**		
P41A/S42A	GACATATACCAAATCGCTGCTGTTGATTCTGCTG	CAGCAGAATCAACAGCAGCGATTTGGTATATGTC
V43A/D44A	ACCAAATCCCTTCTGCTGCTTCTGCTGACAATCT	AGATTGTCAGCAGAAGCAGCAGAAGGGATTTGGT
S45A/A46A	ATCCCTTCTGTTGATGCTGCTGACAATCTATCTG	CAGATAGATTGTCAGCAGCATCAACAGAAGGGAT
D47A/N48A	CTGTTGATTCTGCTGCCGCTCTATCTGAAAAATTG	CAATTTTTCAGATAGAGCGGCAGCAGAATCAACAG
L49A/S50A	GATTCTGCTGACAATGCAGCTGAAAAATTGGAAAG	CTTTCCAATTTTTCAGCTGCATTGTCAGCAGAATC
E51A/K52A	CTGACAATCTATCTGCAGCATTGGAAAGAGAATGG	CCATTCTCTTTCCAATGCTGCAGATAGATTGTCAG
L53A/E54A	AATCTATCTGAAAAAGCGGCAAGAGAATGGGATAG	CTATCCCATTCTCTTGCCGCTTTTTCAGATAGATT
R55A/E56A	TCTGAAAAATTGGAAGCAGCATGGGATAGAGAGCT	AGCTCTCTATCCCATGCTGCTTCCAATTTTTCAGA
W57A/D58A	AAATTGGAAAGAGAAGCGGCTAGAGAGCTGGCTTC	GAAGCCAGCTCTCTAGCCGCTTCTCTTTCCAATTT
R59A/E60A	GAAAGAGAATGGGATGCAGCGCTGGCTTCAAAGAA	TTCTTTGAAGCCAGCGCTGCATCCCATTCTCTTTC
L61A/A62A	GAATGGGATAGAGAGGCGGCTTCAAAGAAAAATCC	GGATTTTTCTTTGAAGCCGCCTCTCTATCCCATTC
S63A/K64A	GATAGAGAGCTGGCTGCAGCGAAAAATCCTAAACT	AGTTTAGGATTTTTCGCTGCAGCCAGCTCTCTATC
K65A/N66A	GAGCTGGCTTCAAAGGCAGCTCCTAAACTCATTAA	TTAATGAGTTTAGGAGCTGCCTTTGAAGCCAGCTC
R55A	TCTGAAAAATTGGAAGCAGAATGGGATAGAGAG	CTCTCTATCCCATTCTGCTTCCAATTTTTCAGA
E56A	CTGAAAAATTGGAAAGAGCATGGGATAGAGAGCTG	CAGCTCTCTATCCCATGCTCTTTCCAATTTTTCAG
W57A	GAAAAATTGGAAAGAGAAGCGGATAGAGAGCTGGC	GCCAGCTCTCTATCCGCTTCTCTTTCCAATTTTTC
D58A	TTGGAAAGAGAATGGGCTAGAGAGCTGGCTTCA	TGAAGCCAGCTCTCTAGCCCATTCTCTTTCCAA
R59A	GGAAAGAGAATGGGATGCAGAGCTGGCTTCAAAG	CTTTGAAGCCAGCTCTGCATCCCATTCTCTTTCC
E60A	GAGAATGGGATAGAGCGCTGGCTTCAAAGAAA	TTTCTTTGAAGCCAGCGCTCTATCCCATTCTC
L61A	GAATGGGATAGAGAGGCGGCTTCAAAGAAAAATCC	GGATTTTTCTTTGAAGCCGCCTCTCTATCCCATTC
A62S	TGGGATAGAGAGCTGTCTTCAAAGAAAAATCC	GGATTTTTCTTTGAAGACAGCTCTCTATCCC
**2. Site-directed mutagenesis (QuickChange): ABCC7**		
W57G	GAAAAATTGGAAAGAGCAGGGGATAGAGAGCTGGC	GCCAGCTCTCTATCCCCTGCTCTTTCCAATTTTTC
W57F	GAAAAATTGGAAAGAGAATTCGATAGAGAGCTGGC	GCCAGCTCTCTATCGAATTCTCTTTCCAATTTTTC
W57Q	GAAAAATTGGAAAGAGCACAGGATAGAGAGCTGGC	GCCAGCTCTCTATCCTGTGCTCTTTCCAATTTTTC
W57L	GAAAAATTGGAAAGAGCACTGGATAGAGAGCTGGC	GCCAGCTCTCTATCCAGTGCTCTTTCCAATTTTTC
W57P	GAAAAATTGGAAAGAGCACCGGATAGAGAGCTGGC	GCCAGCTCTCTATCCGGTGCTCTTTCCAATTTTTC
W57Y	GAAAAATTGGAAAGAGCATACGATAGAGAGCTGGC	GCCAGCTCTCTATCGTATGCTCTTTCCAATTTTTC
W57E	GAAAAATTGGAAAGAGCAGAGGATAGAGAGCTGGC	GCCAGCTCTCTATCCTCTGCTCTTTCCAATTTTTC
W57R	GAAAAATTGGAAAGAGCACGGGATAGAGAGCTGGC	GCCAGCTCTCTATCCCGTGCTCTTTCCAATTTTTC
E60K	GAGAATGGGATAGAAAGCTGGCTTCAAAGAAAAAT	GATTTTTCTTTGAAGCCAGCTTTCTATCCCATTCTC
E60D	GAGAATGGGATAGAGACCTGGCTTCAAAGAAAAAT	ATTTTTCTTTGAAGCCAGGTCTCTATCCCATTCTC
E60S	GAGAATGGGATAGATCGCTGGCTTCAAAGAAAAAT	ATTTTTCTTTGAAGCCAGCGATCTATCCCATTCTC
E60G	GAGAATGGGATAGAGGGCTGGCTTCAAAGAAA	TTTCTTTGAAGCCAGCCCTCTATCCCATTCTC
E60Q	GAGAATGGGATAGACAGCTGGCTTCAAAGAAA	TTTCTTTGAAGCCAGCTGTCTATCCCATTCTC
E60V	GAGAATGGGATAGAGTGCTGGCTTCAAAGAAA	TTTCTTTGAAGCCAGCACTCTATCCCATTCTC
E60Y	AGAGAATGGGATAGATACCTGGCTTCAAAGAAA	TTTCTTTGAAGCCAGGTATCTATCCCATTCTCT
E60W	GAGAATGGGATAGATGGCTGGCTTCAAAGAAA	TTTCTTTGAAGCCAGCCATCTATCCCATTCTC
**3. Inverse PCR-based site-specific deletion: ABCC7**		
NT80/Δ1	CCAATTTTGAGGAAAGGATACAGACAG	CATGGTCGACCGAATTCGGGCCTC
NT80/Δ2	CCTTCTGTTGATTCTGCTGACAATCTA	TCTGGTCCAGCTGAAAAAAAGTTTGG
NT80/Δ3	CTGGCTTCAAAGAAAAATCCTAAACTC	GATTTGGTATATGTCTGACAATTCCAG
NT80/Δ4	TAGGTACCGCGGCCGCGGGGATCC	CTCTCTATCCCATTCTCTTTCCAATTT
**4. Site-directed mutagenesis (QuickChange): ABCC4**		
W64A	GAGTTGCAAGGGTTCGCGGATAAAGAAGTTTTAAG	CTTAAAACTTCTTTATCCGCGAACCCTTGCAACTC
D65A	GTTGCAAGGGTTCTGGGCTAAAGAAGTTTTAAGAG	CTCTTAAAACTTCTTTAGCCCAGAACCCTTGCAAC
K66A	GCAAGGGTTCTGGGATGCAGAAGTTTTAAGAGCTG	CAGCTCTTAAAACTTCTGCATCCCAGAACCCTTGC
E67A	GGGTTCTGGGATAAAGCAGTTTTAAGAGCTGAGAA	TTCTCAGCTCTTAAAACTGCTTTATCCCAGAACCC
