# Supplementary material for: A conserved WXXE motif is an apical delivery determinant of ABC transporter C subfamily isoforms
Source: Cell Struct Funct. 2023 Jan 25;48(1):71–82. doi: 10.1247/csf.22049 (PMC10721954; doi:10.1247/csf.22049)
Supplement: Supplementary file 2 — Supplementary Figures [file csf_48_22049_2.pdf]

Figure legends

Fig. S1. Experimental design for assessing polarized distribution of proteins in HepG2 cells. (A) Predicted membrane topology. ABCC7 and ABCC2 display a comparable membrane topology. Walker A and B motifs and the family signature C present in the NBD1 and NBD2 domains are also indicated. ATS is the apical targeting signal of ABCC2. NT80 of ABCC7 and CLD1 of ABCC2 are followed by MSD1. (B) Polarizerd distribution of ectopically expressed FLAG-ABCC7, FLAG-ABCC2, and ABCC1-HA in HepG2 cells. HepG2 cells develop polarity and form sealed vacuoles between the plasma membranes of two adjacent cells. Canalicular vacuoles and cell perimeters were visualized by labeling with rhodamine-conjugated phalloidin, which demarcated a subplasmalemmal mesh-like structure of filamentous actin (F-actin). Positions of apical vacuoles formed between the juxtaposed polarized cells are indicated with white arrowheads. (C) Effect of overexpression of NT80 on the localization of FLAG-ABCC7 in HepG2 cells. Cells were transiently co-transfected with FLAG-ABCC7 (0.5 μg) and Myc-NT80 (1.0 μg) expression plasmids. (D) Disruption of the apical vacuolar localization of FLAG-ABCC7 by accumulation of cytoplasmically expressed Myc-NT80. Cells were transiently co-transfected as in (C). Myc NT80 (red) and FLAG-ABCC7 (green) are co-stained with anti-HA polyclonal antibodies and anti-FLAG monoclonal antibodies, respectively. Shown are the representative immunofluorescence images. Apparent exppression levels of Myc-NT80 in FLAG-ABCC7 expressing cells are indicated in the right. Scale bars: 20 μm.

Fig. S2. Subcellular distribution of mislocalized FLAG-ABCC7 accompanied by appearance in the dot-like structures. (A) Expression of fluorescent marker proteins of secretory pathway compartments in HepG2 cells. mCherry-Sec61β, LAMP1-RFP, DsRed-Rab5 WT, DsRed-rab7 WT, and DsRed-rab11 WT were separately transfected into HepG2 cells. Fluorescence images were

acquired for each marker protein (red) following staining of F-actin with Alexa Fluor 488–conjugated phalloidin (green). (B) Subcellular distribution of mislocalized FLAG-ABCC7. Plasmids for FLAG-ABCC7 (0.45 μg) and Myc-CLs (0.5 μg) together with plasmids for indicated fluorescent markers (0.05 μg) were transiently transfected into cells. Representative immunofluorescence images of the HepG2 cells transiently expressing FLAG-ABCC7 are shown. Apical vacuoles formed between two juxtaposed cells were identified by staining with Alexa Fluor 350–conjugated phalloidin and indicated using white arrowheads. Scale bars: 20 μm.

Fig. S3. Only Myc-NT80 disturbed the polarized distribution of FLAG-ABCC7 in a competitive manner. (A) Disruption of the apical localization of ABCC7. The indicated combinations of FLAG-ABCC7 and competitor constructions were used to transiently transfect HepG2 cells. Canalicular vacuoles and cell perimeters were visualized with rhodamine-conjugated phalloidin (F-actin). Positions of apical vacuoles formed between the juxtaposed polarized cells are indicated with white arrowheads. Shown are the representative immunofluorescence images. Scale bars: 20 μm. (B) Quantification of the disruption of apical localization of ABCC7. HepG2 cells were co-transfected with the FLAG–ABCC7 plasmid and the indicated competitor plasmid. Localization of ABCC7 was determined in these cells as described, the degree of colocalization of FLAG–ABCC2 integrated into the apical vacuole was categorized into three groups as illustrated. The yellow area represents co-localization that is most similar to the normal condition. The percentage of cells displaying each localization pattern was plotted (horizontal bar plot). Open bar, apical; light gray bar, intermediate; and dark gray bar, cytoplasmic. Polarized cells were counted according to criteria based on the formation of the apical vacuole, and the number of cells is shown in parentheses.

Fig. S4. Identification of an apical localizaion determinant of ABCC7. A series of alanine scanning mutants were generated by replacing the residues 41–66 in the NT80 region of ABCC7 (two residues at a time as indicated) as described. HepG2 cells transiently expressing the wild-type or the indicated mutant were fixed and subsequently used for immunostaining the expressed FLAG-ABCC7 using the anti-FLAG monoclonal antibody. Fluorescence co-localization images were acquired for each ABCC7 variant following staining of the apical vacuoles (indicated by white arrowheads). Shown are the representative merged immunofluorescence images of the transfected cells. Scale bars: 20 μm.

Fig. S5. W^{57} and E^{60} are functionally critical residues. Point mutations were introduced at W^{57} and E^{60} residues of ABCC7 by site-directed mutagenesis. Transient transfection of HepG2 cells with the indicated mutant plasmids and subsequent processing of cells for immunostaining were carried out as described in the legend of Fig. 3. Shown here are the representative immunofluorescence images of the transfected cells expressing the wild-type or the indicated point mutants. Apical vacuoles were visualized with rhodamine–conjugated phalloidin (F-actin) and are indicated using white arrowheads. Scale bars: 20 μm.

Fig. S1

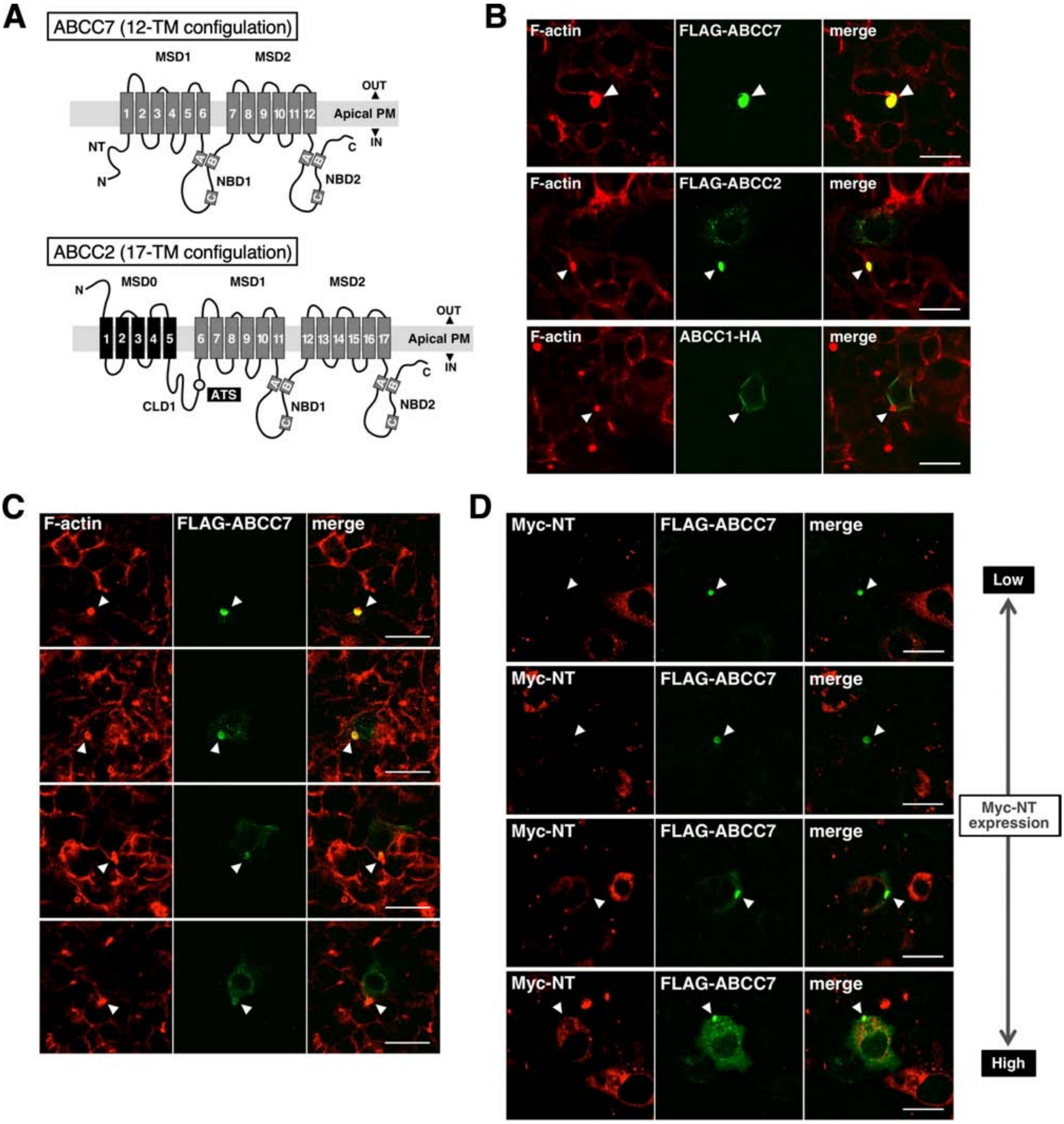
A
ABCC7 (12-TM configulation)
MSD1
MSD2
OUT
IN
Apical PM
NT
N
C
NBD1
NBD2
ABCC2 (17-TM configulation)
MSD0
MSD1
MSD2
CLD1
ATS
B
F-actin
FLAG-ABCC7
merge
FLAG-ABCC2
ABCC1-HA
C
D
Myc-NT
Low
High
Myc-NT expression

Fig. S2

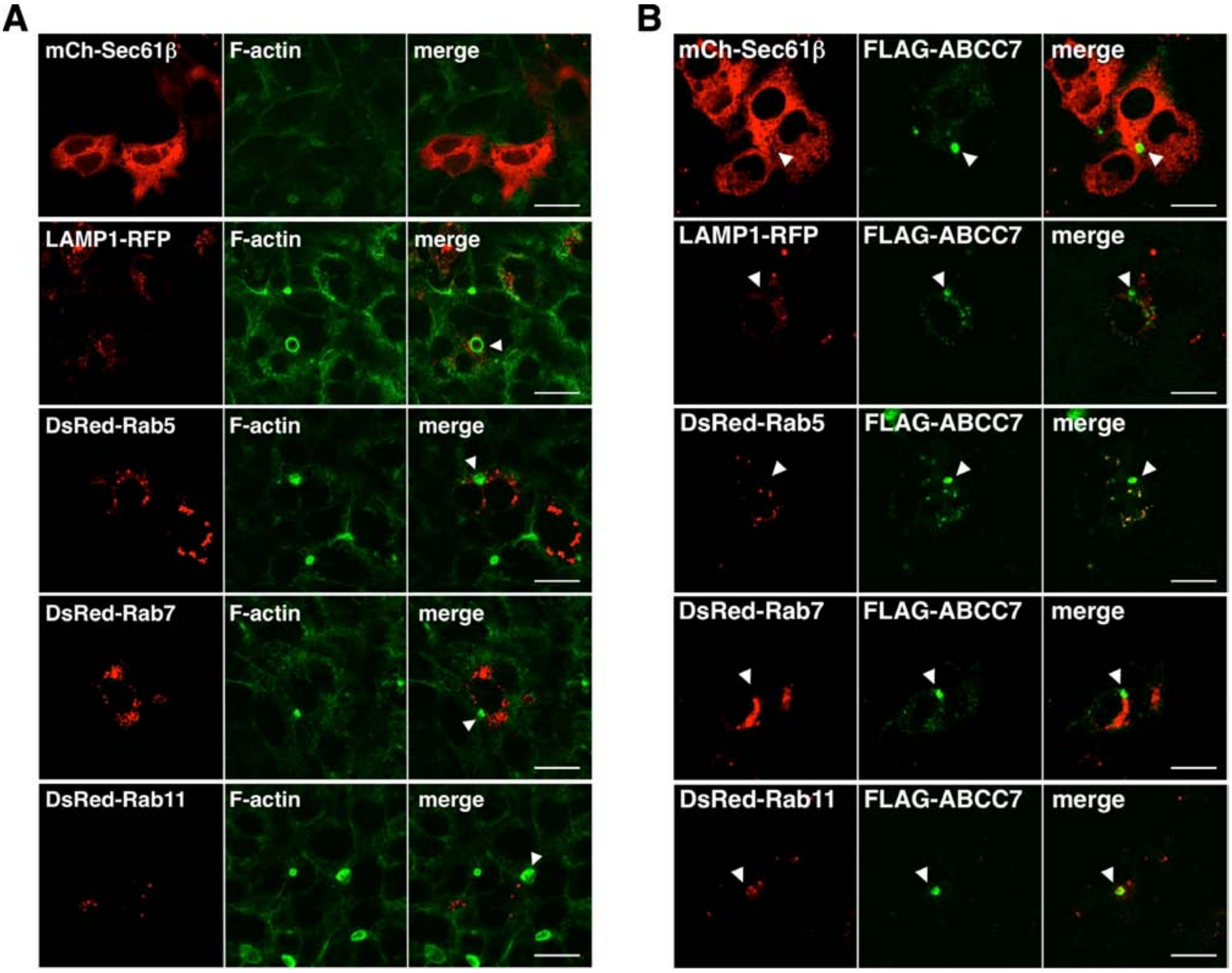
A
mCh-Sec61β
F-actin
merge
LAMP1-RFP
F-actin
merge
DsRed-Rab5
F-actin
merge
DsRed-Rab7
F-actin
merge
DsRed-Rab11
F-actin
merge
B
mCh-Sec61β
FLAG-ABCC7
merge
LAMP1-RFP
FLAG-ABCC7
merge
DsRed-Rab5
FLAG-ABCC7
merge
DsRed-Rab7
FLAG-ABCC7
merge
DsRed-Rab11
FLAG-ABCC7
merge

Fig. S3

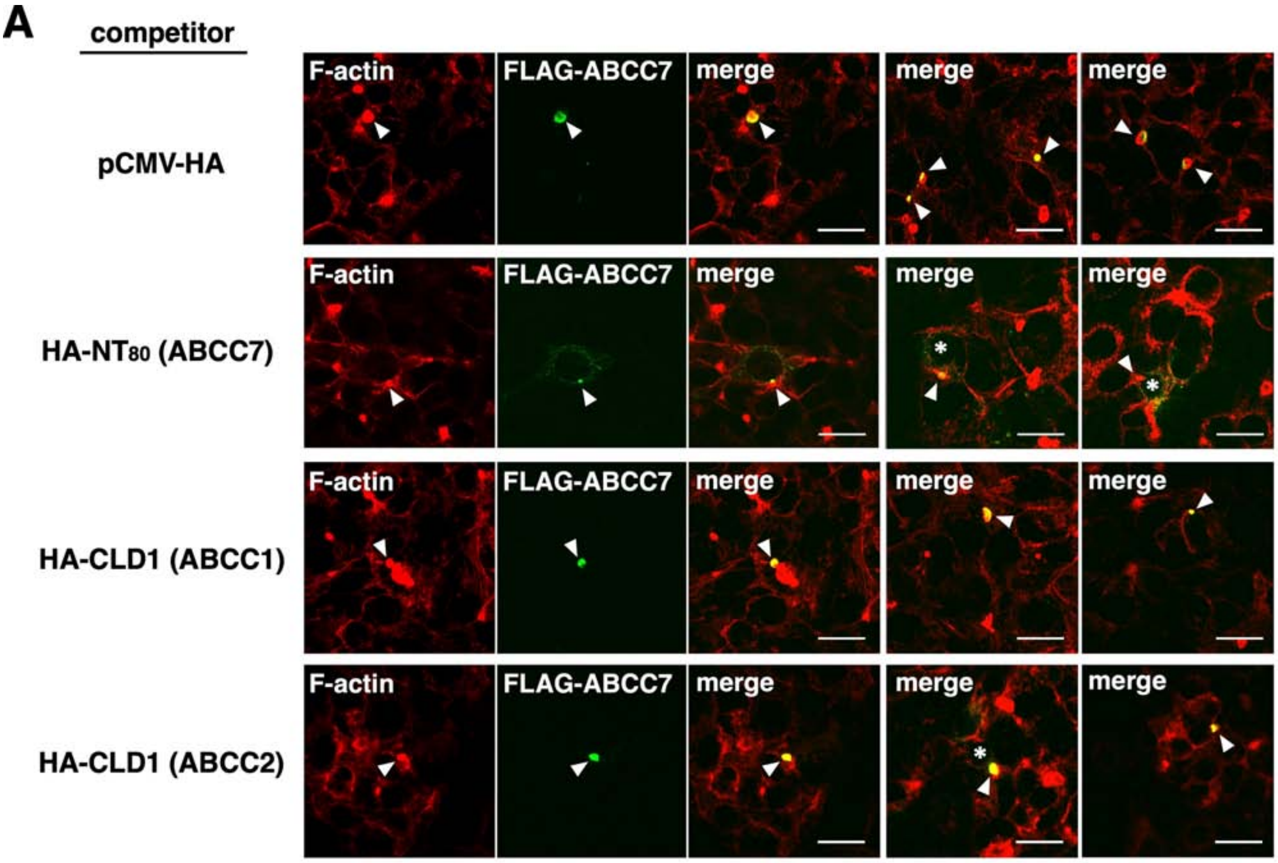
A
competitor
pCMV-HA
HA-NT80 (ABCC7)
HA-CLD1 (ABCC1)
HA-CLD1 (ABCC2)
F-actin
FLAG-ABCC7
merge

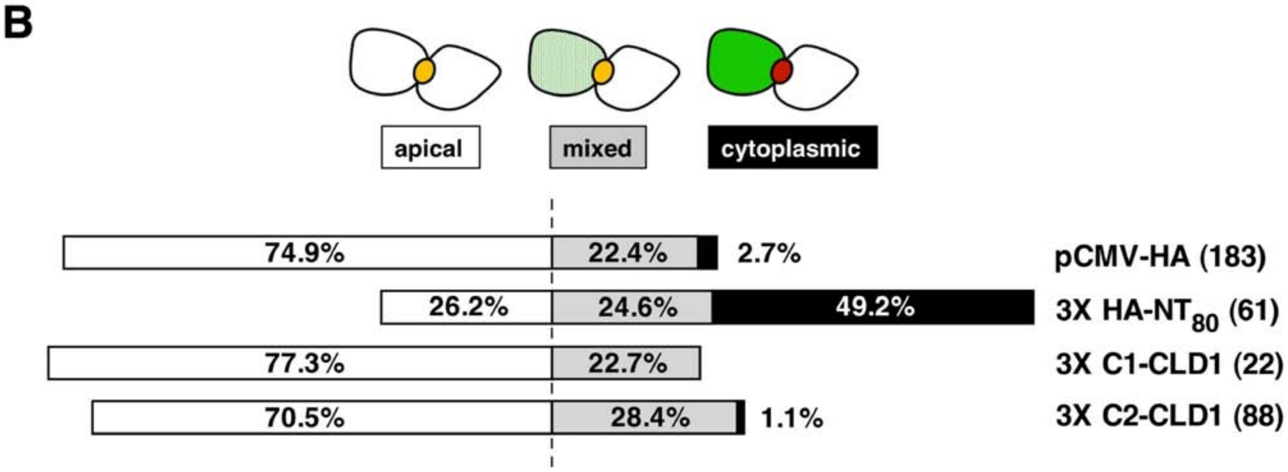
B
apical
mixed
cytoplasmic
74.9%
22.4%
2.7%
pCMV-HA (183)
26.2%
24.6%
49.2%
3X HA-NT80 (61)
77.3%
22.7%
3X C1-CLD1 (22)
70.5%
28.4%
1.1%
3X C2-CLD1 (88)

Fig. S4

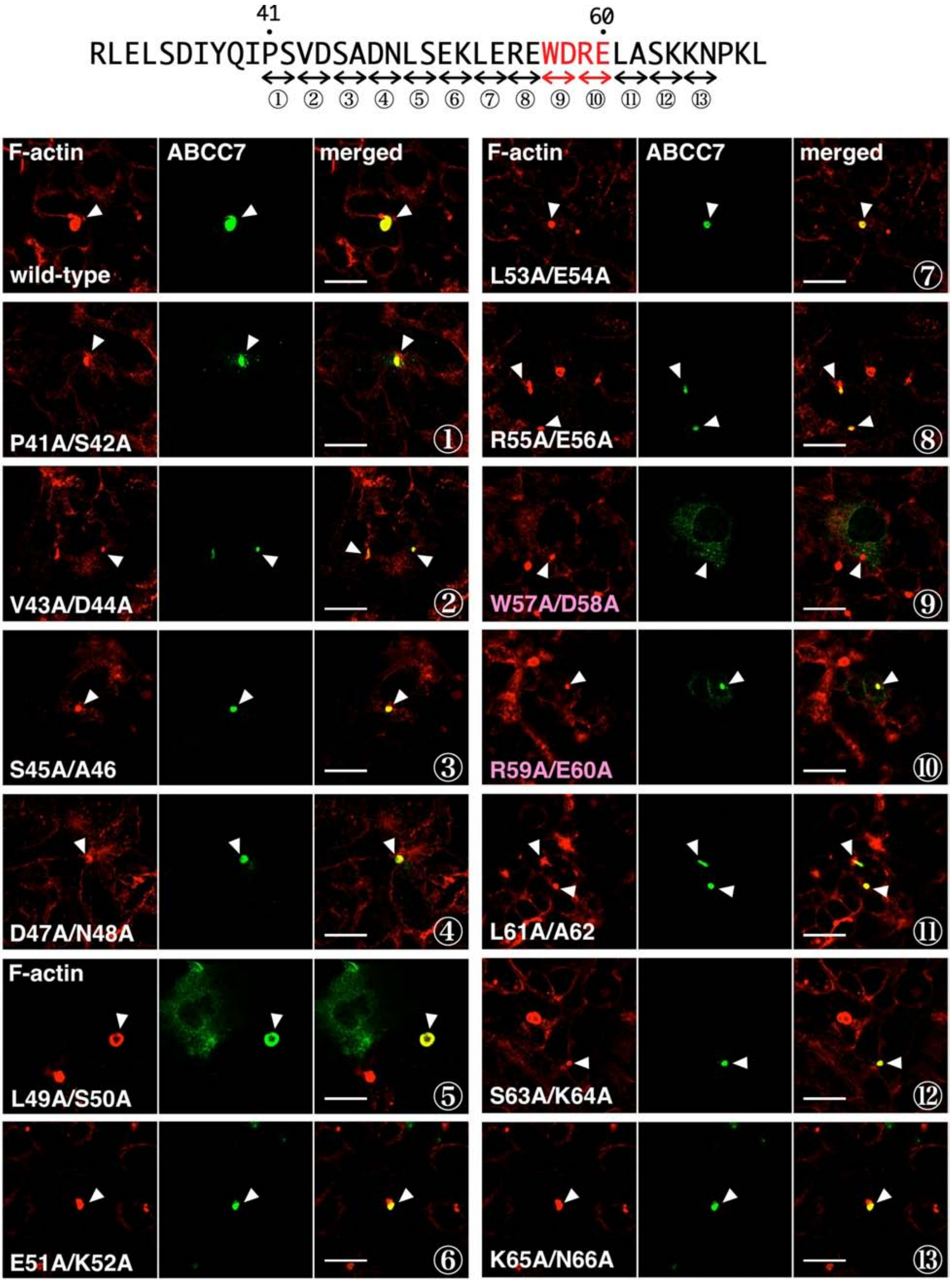
41
60
RLELSDIYQIPSVDSADNLSEKLEREWDRELASKKNPKL
① ② ③ ④ ⑤ ⑥ ⑦ ⑧ ⑨ ⑩ ⑪ ⑫ ⑬
F-actin
ABCC7
merged
wild-type
P41A/S42A
V43A/D44A
S45A/A46
D47A/N48A
L49A/S50A
E51A/K52A
L53A/E54A
R55A/E56A
W57A/D58A
R59A/E60A
L61A/A62
S63A/K64A
K65A/N66A

Fig. S5

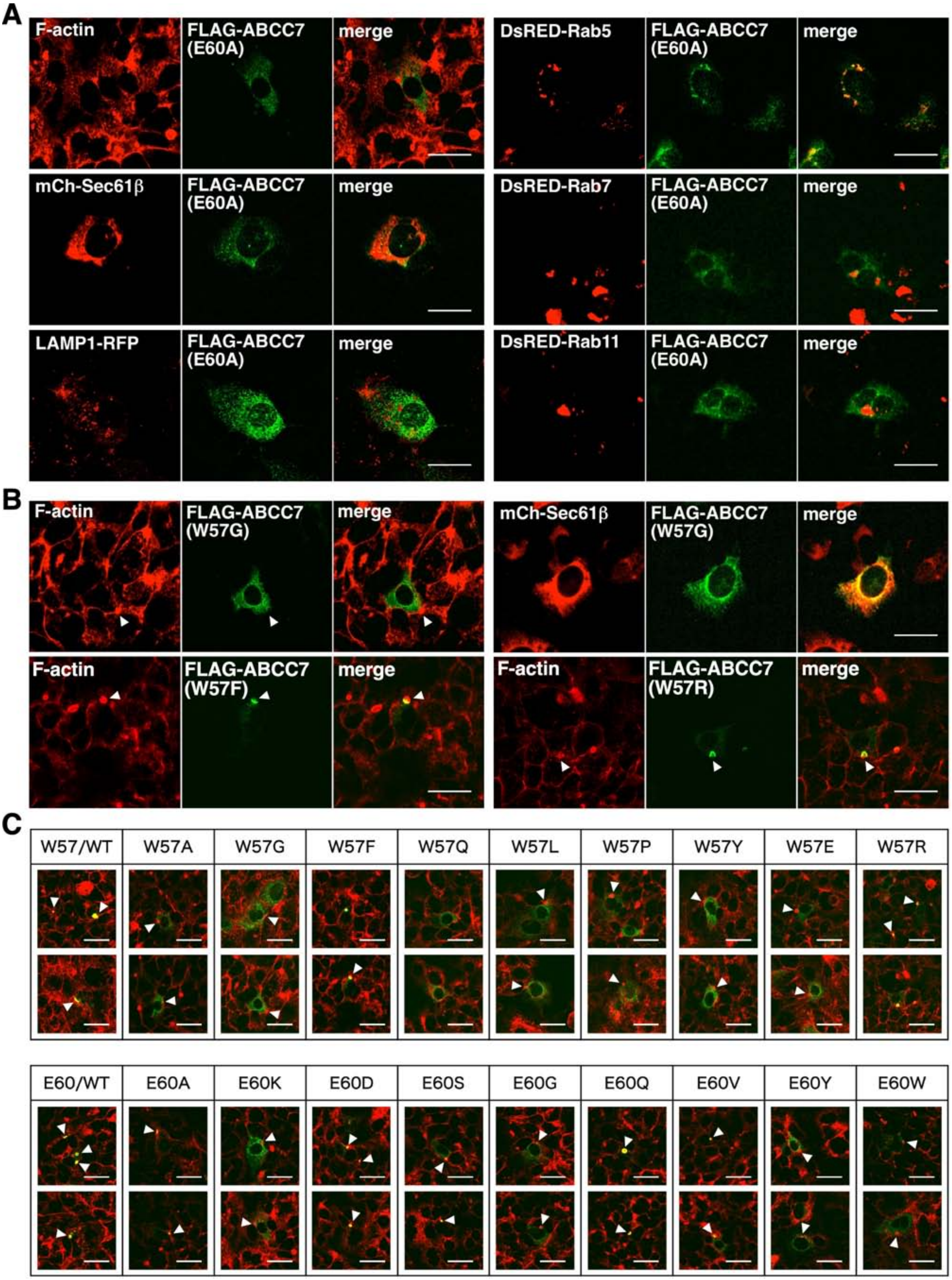
A
F-actin
FLAG-ABCC7 (E60A)
merge
DsRED-Rab5
mCh-Sec61β
DsRED-Rab7
LAMP1-RFP
DsRED-Rab11
B
FLAG-ABCC7 (W57G)
FLAG-ABCC7 (W57F)
FLAG-ABCC7 (W57R)
C
W57/WT
W57A
W57G
W57F
W57Q
W57L
W57P
W57Y
W57E
W57R
E60/WT
E60A
E60K
E60D
E60S
E60G
E60Q
E60V
E60Y
E60W
